# Evaluation of Mechanical compressive strength of cementitious matrix with 12% of IER formulated by modified polymer (NEPS) at different percentages

**DOI:** 10.1038/s41598-020-59482-6

**Published:** 2020-02-12

**Authors:** Atiqa Bekhta, Rachid Hsissou, Ahmed Elharfi

**Affiliations:** 1 Laboratory of Agricultural Resources, Polymers and Process Engineering, Team of Organic Chemistry and Polymers, Department of Chemistry, Faculty of Science, University Ibn Tofail, BP 133, 14000 Kenitra, Morocco; 2 Team of Innovative Materials and Mechanical Manufacturing Process, ENSAM, University Moulay Ismail, B.P. 15290 Al Mansour, Meknes Morocco

**Keywords:** Chemistry, Engineering, Materials science

## Abstract

During this paper, we improved the compressive strength of cementitious matrix based on ion exchanging resin (IER) at 12% and formulated by the modified novolac epoxy polymer surfactant (NEPS) at various percentages (0, 1, 2, 3, 4 and 5%). The results show that the introduction of 1% and 2% of NEPS in the cementitious matrix with 12% of IER increases the compressive strength compared to that of the basic matrix (from 7 to 90 days). However, the formulations 3, 4 and 5% show the compressive strength is less than that of the basic matrix (from 28 to 90 days).

## Introduction

Epoxy polymers cover various industrial areas such as: electronics, coating, inhibition, space construction and radioactive waste^1–5^. Epoxy polymers have many properties, including viscosimetric, thermal, morphological, rheological and mechanical compressive of strength^6–9^. At this time, the most widely employed process for the synthesis of epoxy polymers is the condensation of epichlorohydrin on structures having at least two mobile hydrogen atoms such as diamines, diacids and polyphenols. Also, the oxidation of polyunsaturated compounds and/or the condensation of glycidol with halogenated compounds^10,11^. Novolac epoxy polymers are obtained by the condensation of phenol with formaldehyde in an acid medium^12,13^.

The objective of this paper is to improve the chemical compressive of strength of the cementitious matrix based on ion exchanging resin (IER) at 12% formulated by novolac epoxy polymer surfactant (NEPS) modified at various percentages (1, 2, 3, 4 and 5%)^14–16^. We have studied the properties of mechanical of compressive strength, varying the polymer amounts incorporated in cementitious matrix formulations. This gave us the reflection to introduce the modified novolac epoxy polymer in surfactant form into the cementitious matrix. Besides, the results obtained show an increase in mechanical compressive of strength after 7, 14, 28 and 90 days of confinement compared to the basic matrix.

## Material and Methods

### Ion exchange resin (IER)

Ion exchange resin is a crosslinked macromolecular water insoluble matrix which, upon contact with a solution, can exchange the ions it contains with other ions of the same sign from the solution used in water purification of the reactor vessel Mark TRIGA II^16^. Their physicochemical properties are shown in Table 1.

**Table 1:** Physical and chemical properties of IER.

Physical and chemical properties of IER	
Skeleton	Polystyrene crossed with DVB of gel type
Functional groups	R-SO_3_-
Physical aspect	Dark amber beads, translucent
Ion shape on delivery	H-
Moisture content	51–65% (H^+^ form)
Maximum swelling	Na^+^ -H^+^: 5%
Temperature limit	120 °C
Limit of pH	From 0 à 14
Apparent density	Approximately 800 g/L
Actual density	1.20 (H+ form)
Total exchange capacity	Min 1.7 eq/l (H^+^ form)
**The granulometry of the resin**	
Less than 0.315 mm	0.2 max
0.4 < X < 1.0 mm	80% min
Greater than 1.25 mm	3% max

### Novolac epoxy polymer (NEP)

**Scheme 1:**
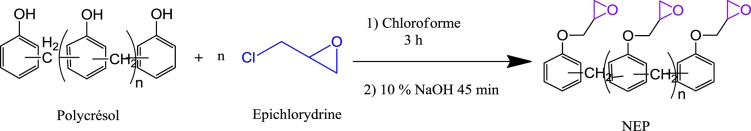
Synthesis of novolac epoxy polymer.

Epoxies are excellent matrices of high performance polymers. The latter are synthesized by polycondensation reaction and are used in several fields such as conditioning of radioactive waste reinforced concrete^17^. The NEP is prepared by condensation of epichlorohydrin with polycresol (hydroxy novolac) polymer in an alkaline medium (Scheme 1).

### Cement

The used CPJ 45 cement has technical characteristics which are conformed to the Moroccan standard NM 10.1.004. The CPJ 45 is a composite Portland cement resulting from the milling of clinker (+70%), the complement of 100% of one or more secondary constituents such as fillers, Pozzolan or fly ash, gypsum to regulate the setting. This cement is also called a hydraulic binder because it has the property of hydrating and curing in the presence of water.

### Molds

**Figure 1:**
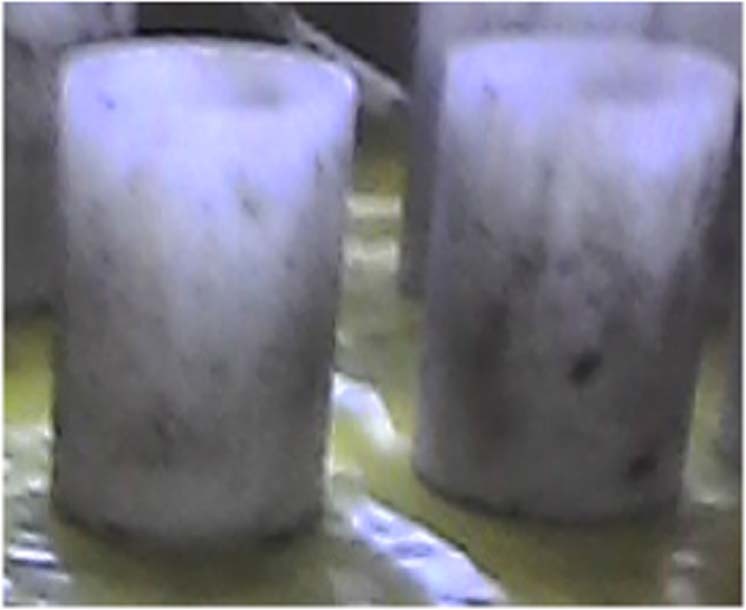
Molds.

The used molds are cylindrical with a diameter of 5 cm and a height of 10 cm, which are illustrated in Fig. 1.

### Mixer

The auto mortar mixer is a device which ensures the mixing of a great homogeneity while reducing the duration of mixing with 30 seconds of time and 285 rpm of speed of mixing.

### Press carver 4350 L

The used press carver 4350 L is manual hydraulic which makes it possible to determine the compressive strength of the mortar from the force measured in view of the surface.

### Preparation of surfactant

**Scheme 2:**
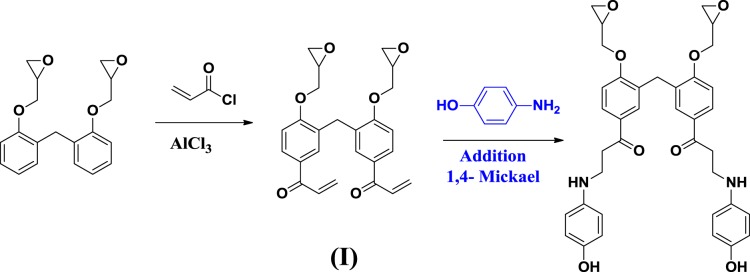
Preparation of modified novolac epoxy polymer surfactant (NEPS).

The synthesis of modified novolac epoxy polymer surfactant (NEPS) based on novolac epoxy polymer (NEP) was carried out in two steps. The first step consists of condensing of 0.004 mol of novolac epoxy polymer with 0.026 mol of acrylic chloride in the presence of Lewis acid (AlCl_3_) by Friedel and Craft acylation reaction with magnetic stirring for 4 hours at 100 °C. Besides, in the second step, 3.014 mol of para-aminophenol were added to the previously product obtained (I) according to the first step by reaction of 1, 4-Mickael addition under magnetic stirring for 3 hours at 70 °C (Scheme 2)^18,19^. All the employed chemicals products were purchased from Aldrich Chemical Co.

### Calculation of compressive strength

The strength is applied to the matrix by two cylindrical metal plates and the reading of the force is either pound or Pound per Square Inch (PSI). Moreover, we calculate the compressive strength (R) from the displayed force value which is expressed by using Eq. 1.

1 $${\rm{R}}=\frac{{\rm{F}}}{{\rm{S}}}$$

With R, F and S denote compressive strength (MPa), force applied (Pound) and surface of the test piece (cm^2^), respectively.

## Methods used

### Fourier transform infrared spectroscopy

The used FTIR spectrometer is a BRUKER Fourier transform spectroscopy. The light beam passes through the sample to a thickness of about 2 μm. The analysis is carried out between 4000 cm^−1^ and 600 cm^−1^.

### Nuclear magnetic resonance

Analyzes of Nuclear magnetic resonance (^1^H NMR and ^13^C NMR) were obtained by using ADVANCE 300 Bruker like apparatus, and the product was solubilized in CDCl_3_. The chemical shifts are expressed in ppm.

### Scanning electron microscope

The scanning electron microscope was used to make photographic images. The observations were carried out on a JEOL-JEC-530 microscope. This technique is based on the use of a beam of electrons accelerated by a fixed potential that excites the surface of the sample.

## Results and Discussions

### Fourier transform infrared spectroscopy

**Figure 2:**
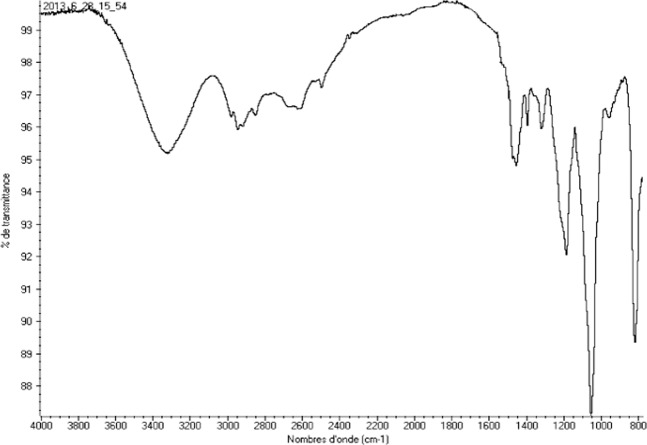
IR spectrum of NEPS modified.

The novolac epoxy polymer surfactant (NEPS) modified was characterized by Fourier transform infrared spectroscopy (FTIR) analysis (Fig. 2). The different bonds NEPS are grouped in Table 2.

**Table 2:** Different bonds of NEPS.

Band υ (cm^−1^)	Attributions
3200	Bond of O–H residual
2920	Bond stretching of CH2
1300-1500-1590	Bond stretching of C=O
1150	Bond stretching of C–O aromatic ethers (Ph–O) and alcohols
1100	Bond of C–C aliphatic
1020	Bond of C–N
815	Bending of CH_2_ (epoxy)

### Nuclear magnetic resonance

**Figure 3:**
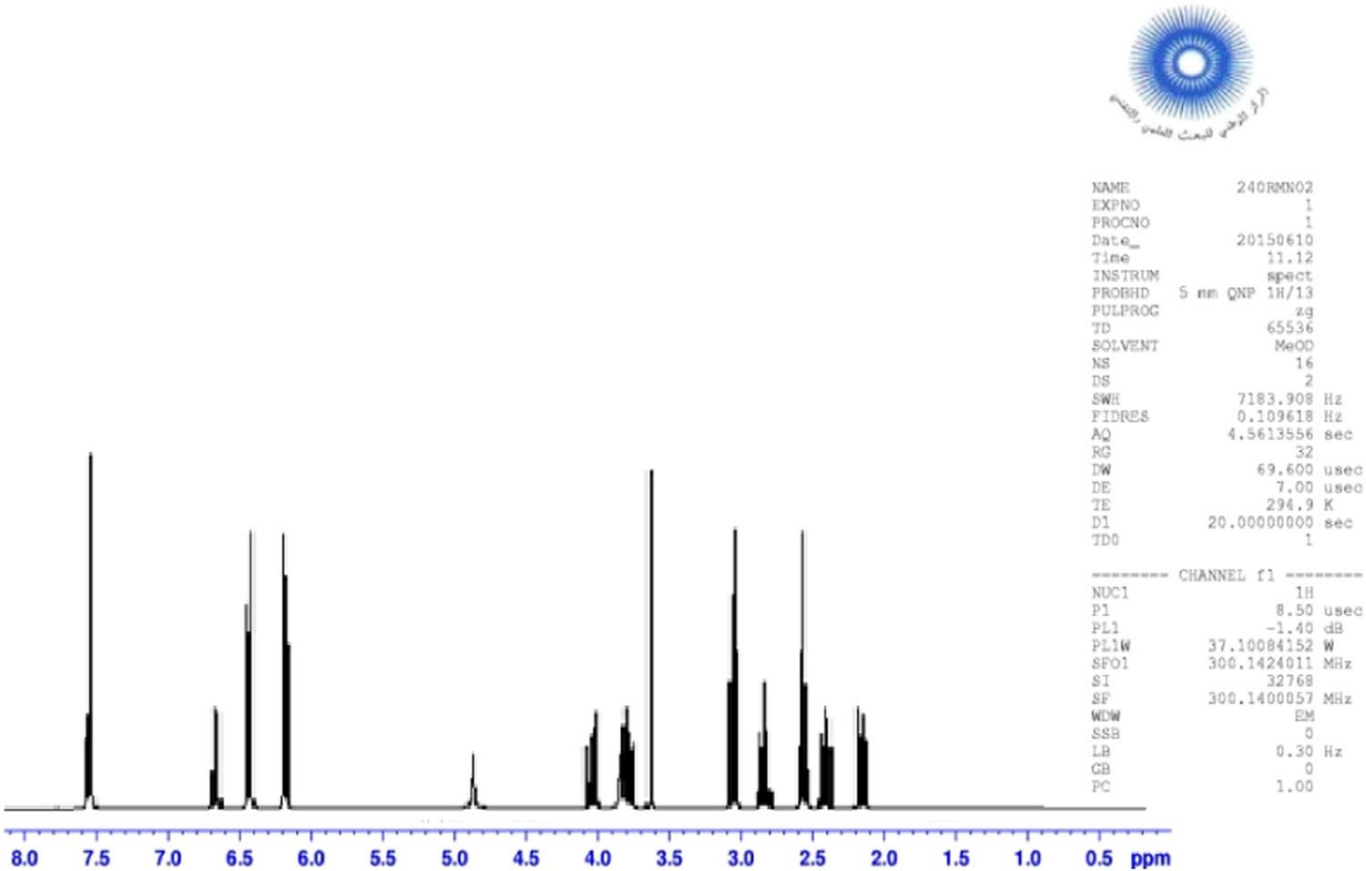
^1^H NMR spectrum of NEPS modified.

**Figure 4:**
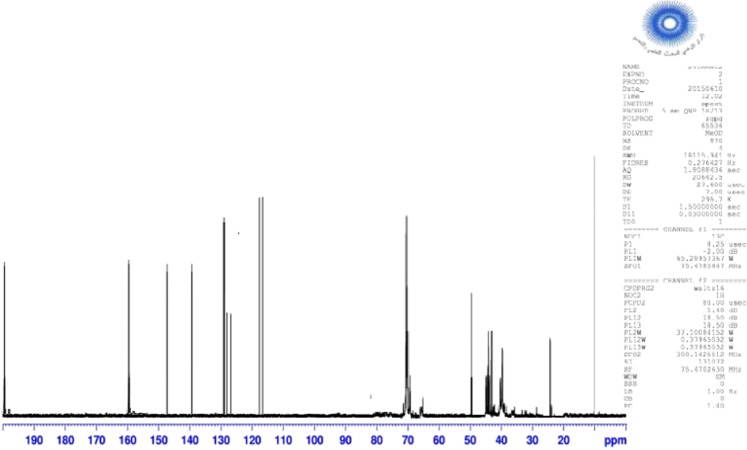
^13^C NMR spectrum of NEPS modified.

The proton and carbon NMR spectrum of the novolac epoxy polymer surfactant modified are shown in Figs. 3 and 4, respectively. The chemical shift (ppm) of proton and carbon of NEPS modified are given as follows.

^1^H NMR (ppm): 2.38–2.63 (m, 2H, CH_2_ of oxiranes); 2.78–3.25 (m, 4H, CH_2_ of methylene); 3.04 (m, 2H, CH of oxiranes); 3.95 (s, 2H, CH_2_ bonded to two benzene); 4.0 (m, 4H, C-NH); 4.20 (dd, 2H, C-H of oxiranes); 5.0 (s, 2H, C-OH) and 6–8 (d, 4H, C-H of benzene).

^13^C NMR (ppm): 26.48 (s, 1C, CH_2_ bonded with two benzene); 36.4–39.2 (s, 4C, CH_2_ of methylene); 44.2 (s, 2C, CH_2_ of the oxirane); 50.7 (s, 2C, CH of oxirane); 69.5 (s, C of CH_2_ bonded with ether); 114.6, 116.9 and 128.6 (s, C of benzene); 140.2 (s, C of benzene bound to NH); 146.5 159.1 (s, C of benzene bound to ether) and 200.0 (s, C of carbonyl).

### Optimization of percentage of NEPS modified

**Figure 5:**
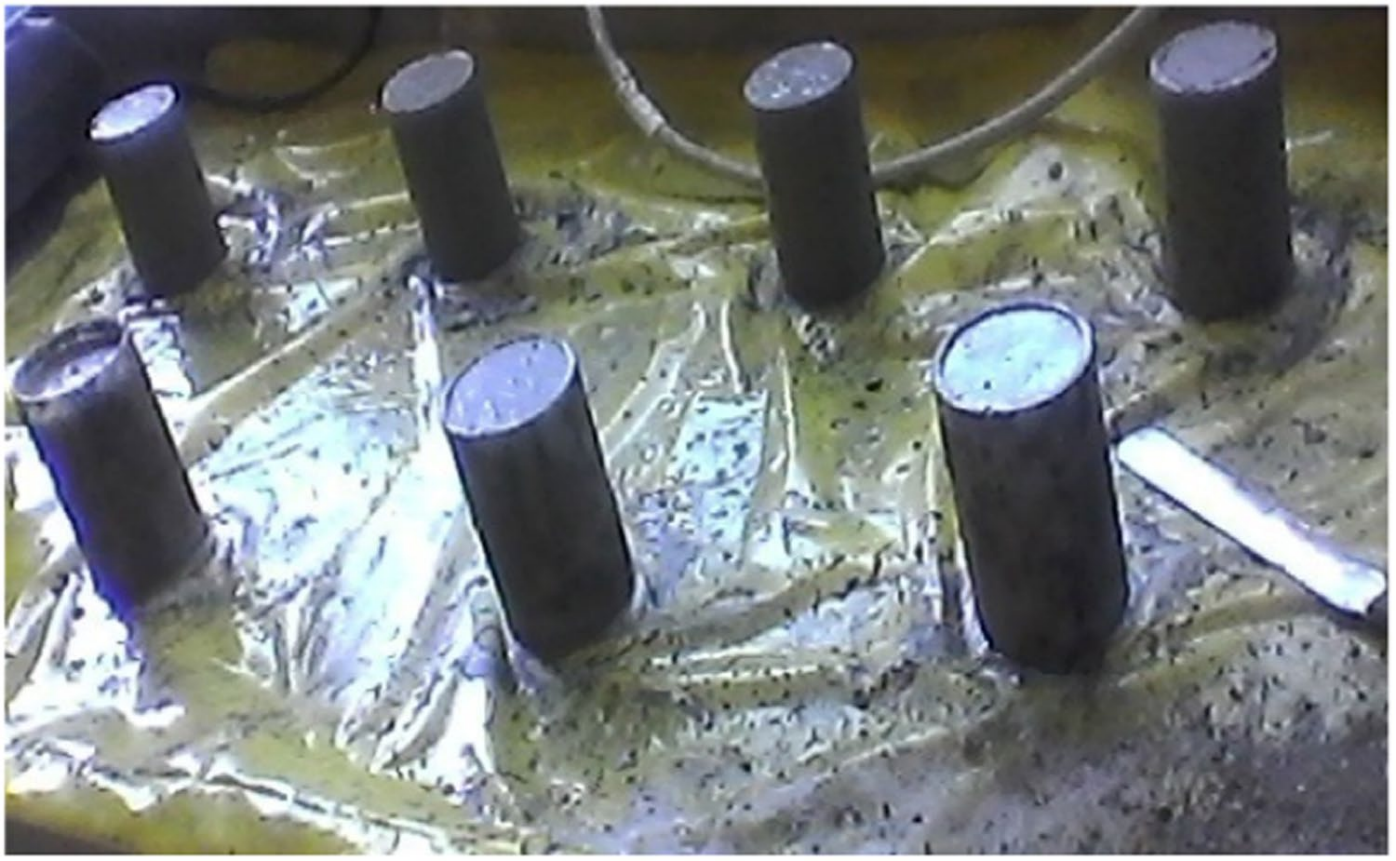
Scheme of confinement matrix.

**Figure 6:**
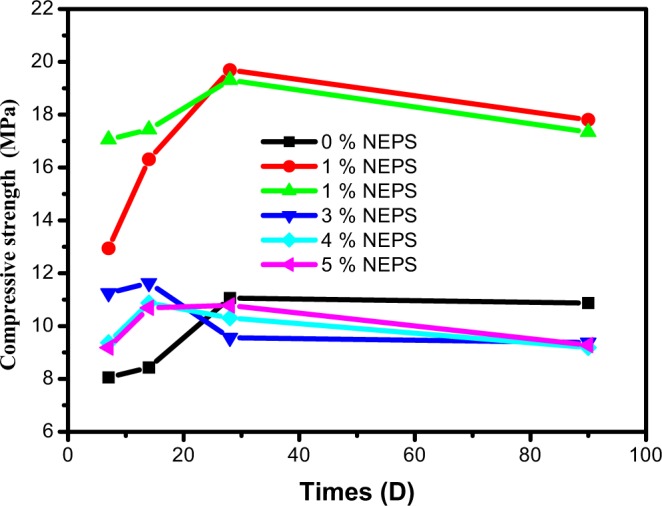
Variation of compressive strength according to time.

The different formulations used for this study are 12% of IER, 67.92% of cement and 20.19% of water. In this study, we tried to introduce the novolac epoxy polymer surfactant (NEPS) modified into the cement matrix so as to improve the compressive strength. The used surfactant polymer is introduced in matrix at different percentages (1, 2, 3, 4 and 5%). Figure 5 shows the confinement matrix. The results of compressive strength for these 7, 14, 28 and 90 days matrix are shown in Table 3. According to these results, we concluded that the introduction of novolac epoxy polymer surfactant modified at various percentages (1, 2, 3, 4 and 5%) into the matrix increases the compressive strength with respect to the base matrix **(**Fig. 6**)**^20^. The matrix of 1% of NEPS modified has a superior compressive strength. Once again, we changed the configuration of the novolac epoxy polymer by modifying it in surfactant form to further improve the compressive strength in the conditioning matrix. Moreover, the compressive strength increases with time for formulations of 1 to 5% novolac epoxy polymer surfactant and for base formulation. Furthermore, the compressive strength of the matrices with the novolac epoxy polymer surfactant of this test is higher than that of the basic matrix up to 28 days, from 28 days to 90 days, where as the tests’ matrix 3, 4 and 5% is less than that of the basic matrix^14^. Besides, the evaluation of these results shows that the introduction of 1% and 2% of NEPS into the IER conditioning matrix increases the compressive strength with respect to the base matrix^16^. They, thus, make it possible to solubilized two immiscible phases. For this reason, we have given a good homogeneity of the conditioned cementitious matrix, as well as good dispersion.

**Table 3:** Compressive strength (CS) of matrix based on NEPS modified (all in MPa).

Times (d)	CS (0% NEPS)	CS (1% NEPS)	CS (2% NEPS)	CS (3% NEPS)	CS (4% NEPS)	CS (5% NEPS)
7	8.06	12.94	17.0625	11.25	9.375	9.187
14	8.43	16.31	17.44	11.63	10.875	10.69
28	11.06	19.69	19.31	9.56	10.31	10.78
90	10.87	17.81	17.34	9.38	9.187	9.28

### Scanning electron microscopy

**Figure 7:**
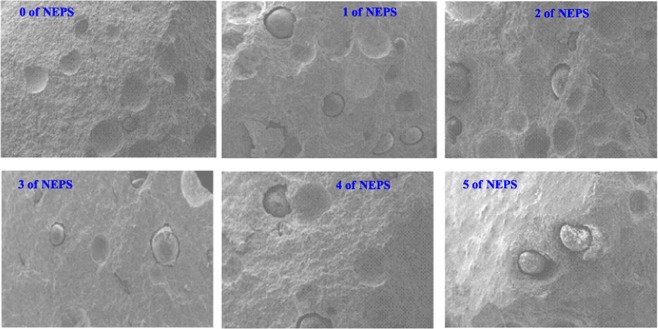
Micrographs of different formulations prepared (0, 1, 2, 3, 4 and 5%).

The dispersion of ions exchanging resin (IER) in the cementitious matrix formulated by novolac epoxy polymer surfactant at various percentages is presented in Fig. 7. The different formulations with addition of 0 to 5% of NEPS modified are analyzed by the scanning electron microscopy (SEM). According to SEM micrographs observations, the cementitious matrix formulated by NEPS clearly show the spherical IER loads on the analyzed surfaces^21,22^. The addition of 1% NEPS exhibits better dispersion, this confirms the higher compressive strength.

## Conclusion

Several research studies have been conducted so as to determine which formulation has better compressibility resistance than the IER conditioning base formulation. Previous studies have evaluated the impact of the novolac epoxy polymer surfactant (NEPS) in different physical states on the formulation. The objective of this study was to improve the compressive strength of the containment matrix by setting the percentage of IER at 12%, the percentage of water at 20.19% and cement at 67.92%, and the introduction of the novolac epoxy polymer surfactant (NEPS) to different percentages (1, 2, 3, 4 and 5%). Besides, the results obtained in this study showed an increase in the compressive strength after 7, 14, 28 and 90 days of confinement with respect to the base formulation. In addition, the introduction of novolac epoxy polymer surfactant into formulation allowed an improvement in the compressive strength of 1% and 2% matrix of the NEPS and good homogeneity of the conditioned cementitious matrix, as well as good dispersion of IER in our formulations on the other hand.
